# Genetic Predisposition to Cancer

**DOI:** 10.1038/sj.bjc.6602883

**Published:** 2006-01-24

**Authors:** F Eisinger

**Affiliations:** 1 CRLCC Institut Paoli-Calmettes, Marseille, France

The US Surgeon General's *‘Family History Initiative’*, launched in 2004, underlines increasing public and professional awareness of the importance of knowing the health history of your close relatives and the benefits this can bring to the health care of family members. Advances in human genetics have given substance to the old ‘experiential’ knowledge of health problems that run in families and the risks of being affected by the same condition. However, this field is not only the province of medical and biological research but is also a minefield of ethical concerns, issues for the wider society, and potential adverse psychological effects on individual *patients* and families. There is a fuzzy border between research and care, with potentially huge cost implications. Inherited cancer syndromes can well be regarded as an example of so-called ‘post normal science’ where facts are uncertain, values in dispute, stakes high, and decisions urgent.

*‘Genetic Predisposition to Cancer’* is welcome, for many surveys show that family and general practitioners and even some specialists have a serious lack of knowledge in this area. The editors of this book are among the world leaders in the field of inherited cancer syndromes and therefore ideally suited to produce this overview.

After a clear and valuable glossary that might, however, have merited more discussion of key issues such as the difference between polymorphism and mutation, the book is divided in five main parts: Basic principles, Inherited cancer syndromes, Chromosome fragility and Gorlin syndrome, Common cancers, and Psychological, ethical and organisational issues. All things considered, this book is, as claimed by the authors, really comprehensive and nearly exhaustive.

The first part gives a concise and valuable overview, even though some articles were probably written 2 or 3 years before. The contribution on genetic linkage and association studies is brilliant and will certainly be useful for both students and academic staff. The second part, ‘Inherited cancer syndromes’, deals with established gene-based syndromes. However, it is not clear why renal cell carcinoma is included in this part while less-common cancers such as pancreatic cancer are placed in the fourth part, ‘Common cancers’. The issue of classification is a critical and difficult. Part two is almost entirely gene-based (*RB1, NF1* and *NF2, MEN1, MEN2, TP53,* and *PTEN*) with the exception of the discussion on renal tumours (both childhood and adulthood), which is organ-based. An organ-based classification is the main basis of the fourth part and leads to some redundancy, for example, with *BRCA2*, which is quoted in Familial breast cancer, Familial ovarian cancer, and Familial prostate cancer. However, despite these minor criticisms, the book is well organised and each chapter is extensively illustrated with figures, tables, algorithms, or clinical pictures, many of which are very well chosen and enlightening. Another very useful feature is the ‘Key Points’ section at the end of each chapter.

This book can be highly recommended for its numerous strengths, but it does suffer from some imbalance between biological and clinical discussion. For example, Chapter 26, Familial Prostate Cancer and its Management, devotes five pages to an extensive description of segregation analysis, linkage analysis, and known genes that are *potentially* involved, before going on to mention that ‘a hint of linkage from one or more collections was subsequently not confirmed…’. Elsewhere, the issue of screening is poorly described, with no mention of the impact of treatment on quality of life (incontinence and sexual dysfunction should be set against the expected increase in life expectancy due to early diagnosis). The lack of discussion of clinical management may perhaps leave clinicians and practitioners uneasy with what is a really valuable scientific overview. There is a genetic and biological perspective throughout the book, exemplified by the use of geneticists' terminology: ‘gene highly penetrant’ where an epidemiologist might have written ‘a high lifetime cumulative risk’ and a clinician something like ‘makes the occurrence of the disease very likely’. Ethical concerns might have merited more analysis (in the case of disclosure particularly), but readers who want more detail will find some interesting references at the end of that article.

*Genetic Predisposition to Cancer* is not a book that can be read at one sitting from cover to cover, but it will be very useful as a reference; particularly, the first three parts, Basic principles, Inherited Cancer Syndromes and Chromosome fragility syndromes, and the Gorlin Syndrome. It will be a particularly valuable reference for students and clinicians who want an overview of this fast moving field. It can be recommended as the root and branches of a ‘knowledge tree’ whose leaves (update information) will have to be found in scientific journals.

